# Role of the activation peptide in the mechanism of protein C activation

**DOI:** 10.1038/s41598-020-68078-z

**Published:** 2020-07-06

**Authors:** Bosko M. Stojanovski, Leslie A. Pelc, Enrico Di Cera

**Affiliations:** Edward A. Doisy Department of Biochemistry and Molecular Biology, Saint Louis University School of Medicine, St. Louis, MO 63104 USA

**Keywords:** Biological techniques, Biochemistry, Biophysical chemistry, Enzyme mechanisms, Enzymes, Proteins, Proteolysis

## Abstract

Protein C is a natural anticoagulant activated by thrombin in a reaction accelerated by the cofactor thrombomodulin. The zymogen to protease conversion of protein C involves removal of a short activation peptide that, relative to the analogous sequence present in other vitamin K-dependent proteins, contains a disproportionately high number of acidic residues. Through a combination of bioinformatic, mutagenesis and kinetic approaches we demonstrate that the peculiar clustering of acidic residues increases the intrinsic disorder propensity of the activation peptide and adversely affects the rate of activation. Charge neutralization of the acidic residues in the activation peptide through Ala mutagenesis results in a mutant activated by thrombin significantly faster than wild type. Importantly, the mutant is also activated effectively by other coagulation factors, suggesting that the acidic cluster serves a protective role against unwanted proteolysis by endogenous proteases. We have also identified an important H-bond between residues T176 and Y226 that is critical to transduce the inhibitory effect of Ca^2+^ and the stimulatory effect of thrombomodulin on the rate of zymogen activation. These findings offer new insights on the role of the activation peptide in the function of protein C.

## Introduction

The clotting enzyme thrombin performs procoagulant, prothrombotic and pro-inflammatory roles in the blood that are mediated by cleavage of fibrinogen and PAR1^1^. In addition, and somewhat paradoxically, thrombin functions as a potent inhibitor of coagulation by activating the zymogen protein C and producing an enzyme itself endowed with diverse physiological roles as a natural anticoagulant and anti-inflammatory factor^2^. Cleavage of protein C by thrombin alone is extremely inefficient and requires the intervention of the endothelial cofactor thrombomodulin that boosts the *k*_cat_/*K*_m_ for the interaction > 1,000-fold, mainly by enhancing *k*_cat_^3^. Importantly, the thrombin-thrombomodulin complex has exclusive activity toward protein C and no appreciable activity toward fibrinogen and PAR1 due to occupancy of exosite I by the soluble EGF domains of thrombomodulin^4^. Activated protein C inactivates cofactors Va and VIIIa with the assistance of protein S, down regulates the amplification and progression of the coagulation cascade and maintains patency of the capillaries^5,6^. As an anti-inflammatory and cytoprotective agent, activated protein C signals through PAR1 and PAR3 in ways that differ completely from thrombin’s activation mechanism and reduces cellular damage following ischemia/reperfusion of the brain, heart, lungs and kidneys, as well as sepsis^7^.

The protein C pathway is highly relevant to human pathophysiology^5^. For example, deficiency of protein C is linked to often fatal neonatal purpura fulminans^8^ and mild deficiency^9^ or mutations that compromise activation of protein C^10^ cause venous thromboembolism. On the other hand, abnormally low levels of activated protein C are associated with life threatening conditions such as atherosclerosis, stroke, sepsis, and disseminated intravascular coagulation^11–14^.

The mechanism of protein C activation has intrigued investigators for decades but remains incompletely understood. Why is cleavage of protein C by thrombin so inefficient in the absence of thrombomodulin? This property is at odds with thrombin being one of the most proficient proteases of the trypsin family, capable of cleaving fibrinogen and PAR1 at rates that are almost diffusion limited^1,15^. How does thrombomodulin achieve its cofactor effect? Is the effect on thrombin, protein C or both? Previous studies have supported paradigms emphasizing the action of thrombomodulin on thrombin^4,16–23^. For example, removal of potential electrostatic clash between acidic residues in the activation domain of protein C and negatively charged regions on the thrombin surface has been invoked as an important component of the thrombomodulin effect^17,24^. This proposal is difficult to reconcile with a number of observations: increasing the ionic strength of the solution does not oppose but actually favors protein C activation by thrombin^25^; the structure of thrombin bound to a fragment of the activation peptide of protein C documents no clash between D167 at the P3 position of protein C and negatively charged residues around the active site of the enzyme^25^; the same observation is reported by the structure of thrombin bound to a fragment of PAR1 that also bears an acidic residue at the P3 position and yet is the most specific physiological substrate of thrombin^26^. The claim that thrombomodulin causes large conformational changes in thrombin^17–19,24,27^ remains controversial^4, 21,23,28,29^. Under physiological conditions, thrombin is mostly bound to Na^+^ and in a rigid conformation according to recent NMR^30^and rapid kinetics^22^studies, thus leaving little room for large conformational transitions. Available structures of thrombin bound to fragment EGF456 of thrombomodulin^4,23^ are practically identical to the free, physiologically dominant E form of the enzyme^22,31,32^. Although these structures have been crystalized with peptidyl inhibitors bound in the catalytic cleft, which may have precluded detection of any conformational changes induced by thrombomodulin binding, there is no evidence of such changes from the analysis of the hydrolysis of small substrates^21,33^. Other studies have supported a more realistic scenario where the conformation of protein C plays an important role in the activation mechanism. The effect of thrombomodulin is mimicked at least in part by mutations of thrombin^24,34–36^ but also of protein C^37,38^. Ca^2+^ binding to the protease domain of protein C inhibits activation in the absence of thrombomodulin, but stimulates the same reaction in the presence of cofactor^39–41^. Even more compelling is the fact that thrombomodulin enhances the rate of diffusion (*k*_on_) of protein C within the active site of thrombin^29^, a parameter that depends on properties of the enzyme and substrate.

Two recent significant developments in the field have renewed interest in the mechanism of protein C activation. The active site Ser has been studied for years for its role in catalysis^42,43^, but has recently emerged as a major transducer of allosteric effects in the trypsin fold^44^. The role of S195 is manifested through subtle rearrangements of the OH group, without the need for large conformational transitions of the entire active site. Likewise, the Arg residue at the site of cleavage has been considered for years a passive component of zymogen activation^42,45,46^, especially of enzyme cascades^47,48^, but its constitutive exposure to solvent necessary for proteolytic attack has been questioned^49^. Specifically, mutagenesis experiments indicate that several acidic residues (*i.e.,* D167, D172) around the scissile bond interact with R169 at the site of activation and partially protect it against proteolytic cleavage by thrombin^25,39,50,51^. Binding of thrombomodulin is believed to induce conformational changes around the site of activation in protein C that improve accessibility of R169 for effective proteolytic attack^25^. An intriguing new paradigm has emerged for cofactor-assisted interactions between trypsin-like zymogens and proteases that is directly relevant to the mechanism of protein C activation. The cofactor optimizes the orientation of the active site Ser of the enzyme and exposes the Arg residue in the activation domain of substrate.

**Figure 1:**
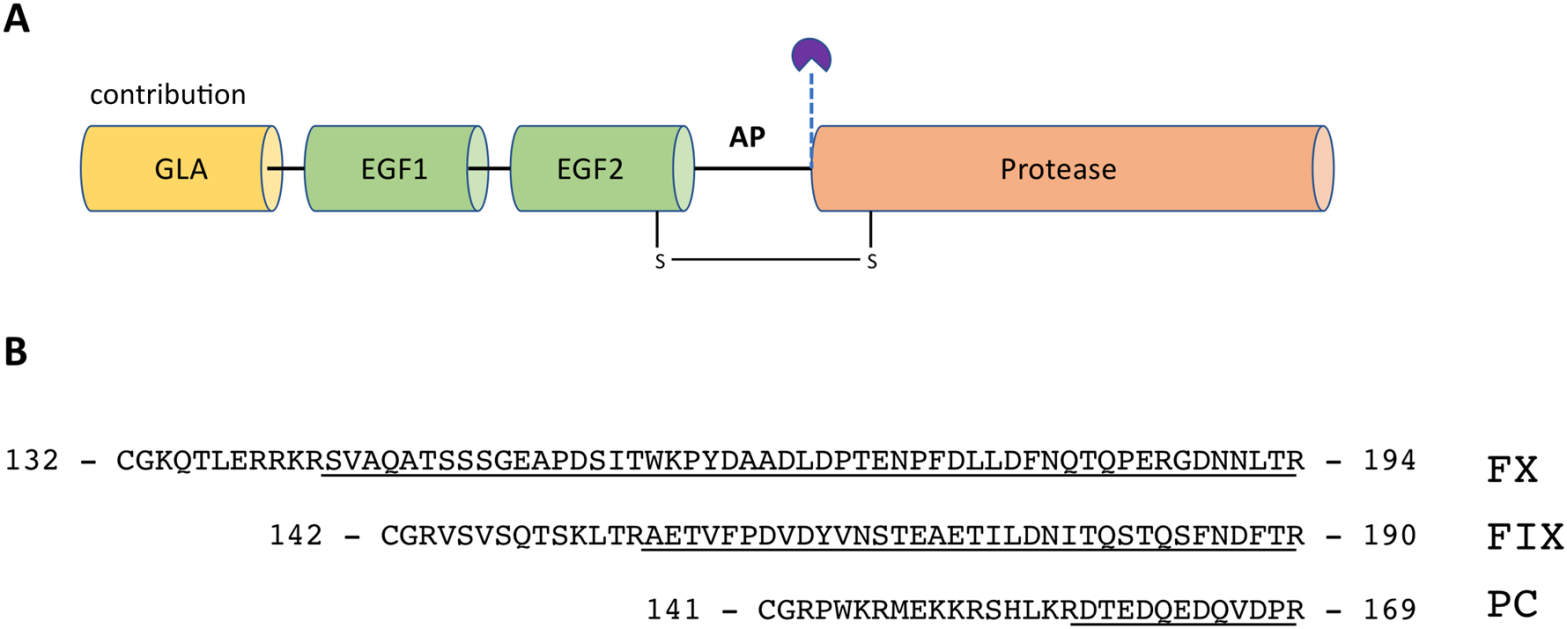
(**A**) Schematic representation of the modular domain assembly of protein C (PC) with the site of thrombin cleavage (dashed line) located in the activation peptide (AP). Identical domain assemblies also characterize the structural architecture of closely related vitamin K-dependent proteins such as factor VII (FVII), factor IX (FIX), and factor X (FX). (**B**) The activation peptides of human FX, FIX, and PC. Shown is the sequence that stretches from the scissile bond Arg to the conserved Cys that forms a disulfide link between the EGF2 and protease domains. Underlined are the residues that comprise the activation peptide, while the remaining  ones are located in the predominantly basic linker that connects the activation peptide with the EGF2 domain.

Protein C shares an identical modular domain assembly (Fig. 1A) with factor VII, factor IX, and factor X including a γ-carboxyglutamate (GLA) domain responsible for interaction with membrane surfaces, two epidermal growth factor (EGF1 and EGF2) domains that primarily serve as spacers and a protease domain which hosts the active site^16,52–59^. With the exception of factor VII, all of the foregoing zymogens contain an activation peptide between the EGF2 and protease domains^54,55,57^. Proteolytic removal of the activation peptide during zymogen activation triggers structural changes in the protease domain that are responsible for organization of the active site^16^. Interestingly, this region contains a peculiar clustering of acidic amino acids that creates a strong negative environment around the site of activation. In fact, half of all amino acids in the activation peptide of protein C have acidic side chains, localized in close proximity to the scissile bond R169-L170 that is cleaved by thrombin during zymogen activation. Because of the short length of the activation peptide, the acidic cluster of amino acids is also proximal to a cluster of basic residues located in a linker that connects the activation peptide with the EGF2 domain. Overall, the peculiar clustering of acidic and basic residues creates a strong dipolar environment around the activation peptide region which prompted us to evaluate its propensity for intrinsic disorder and to characterize the contribution of charged residues toward the activation rate of protein C.

## Results

### Intrinsic disorder propensities

**Figure 2:**
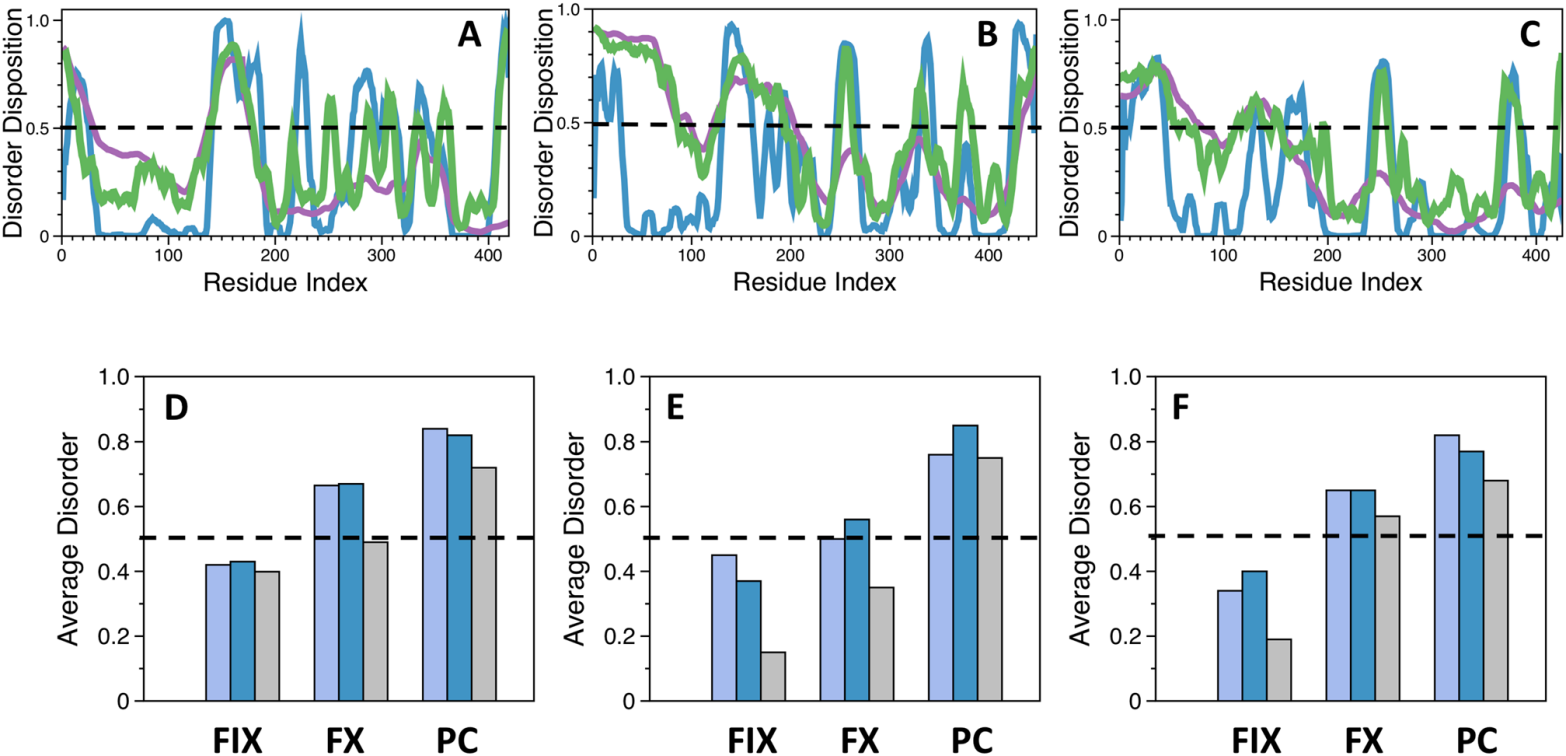
Evaluation of the intrinsic disorder propensity of the amino acid sequences of human **(A)** protein C (PC), **(B)** factor X (FX), and **(C)** factor IX (FIX) analyzed with the VLXT (blue), VL3 (purple) and VSL2 (green) algorithms from the PONDR family of programs. Average disorder scores for the sequences of the activation peptide (purple), the activation peptide and the basic linker that connects it to the EGF2 domain (blue), and the P12-P12′ residues (gray) obtained from analyses with the **(D)** VSL2, **(E)** VLXT, and **(F)** VL3 algorithms. Unprimed and primed numbers respectively denote amino acids located to the N- and C- termini of the scissile bond Arg at the P1 position. Scores were calculated from analysis of the entire amino acid sequence as described in Methods.

Figure 1B lists the amino acid sequences that constitute the activation peptide segment of different human vitamin-K dependent proteins. Significant differences in length, charge distribution and glycosylation exist among these segments, implicating an evolutionary divergence from a common ancestor enzyme. Among the three zymogens, protein C has the shortest activation peptide with only 12 amino acids, while those of factor IX (35 residues) and factor X (52 residues) are significantly longer. The activation peptide segments of factor IX and factor X are also glycosylated^60–62^ and, at least in factor X, the sugar moieties have an adverse effect on the rate of activation^63^and are responsible for extending the zymogen’s half-life in the circulation^64–66^. No glycosylation sites exist in the activation peptide of protein C, but this region features a disproportionately high number of charged residues. Indeed, 55% of all amino acids in the sequence comprising the activation peptide and the basic linker that connects it to the EGF2 domain have either acidic or basic side chains. The total number of charged residues in the analogous segment of factor X and factor IX corresponds to 30% and 22%, respectively. The peculiar localization of charges in protein C is often found in intrinsically disordered regions^67^, which prompted us to evaluate the disorder propensity of the amino acid sequence. For these analyses, we used three algorithms from the PONDR family of programs. Amongst these, the VSL2 algorithm^68^is one of the most accurate stand-alone disorder predictors, VLXT^69^ has high sensitivity to local sequence peculiarities and is often used for identifying disorder-based interaction sites, and VL3^70^ provides accurate evaluation of long disordered regions. A score > 0.5 predicts that a given residue is localized in part of the sequence that tends to be disordered, while the opposite holds true for scores < 0.5. Analysis of the sequence of human protein C predicts that the longest and most disordered region stretches from residues 140 to 180 (Fig. 2A). This region contains the extended sequence around the scissile bond that is cleaved during zymogen activation and includes the basic linker, activation peptide and the 20-loop of the serine protease domain localized upstream of the site of activation. Other regions with notable propensity for disorder include the GLA domain and various loops that are part of the serine protease domain such as the flexible autolysis loop. In contrast, the EGF1 and EGF2 domains are relatively ordered. Extension of this approach to the sequence of human factor IX and factor X (Fig. 2B-C) shows that the activation peptide has the highest degree of disorder in protein C, followed by factor X and factor IX (Fig. 2D-F).

**Figure 3:**
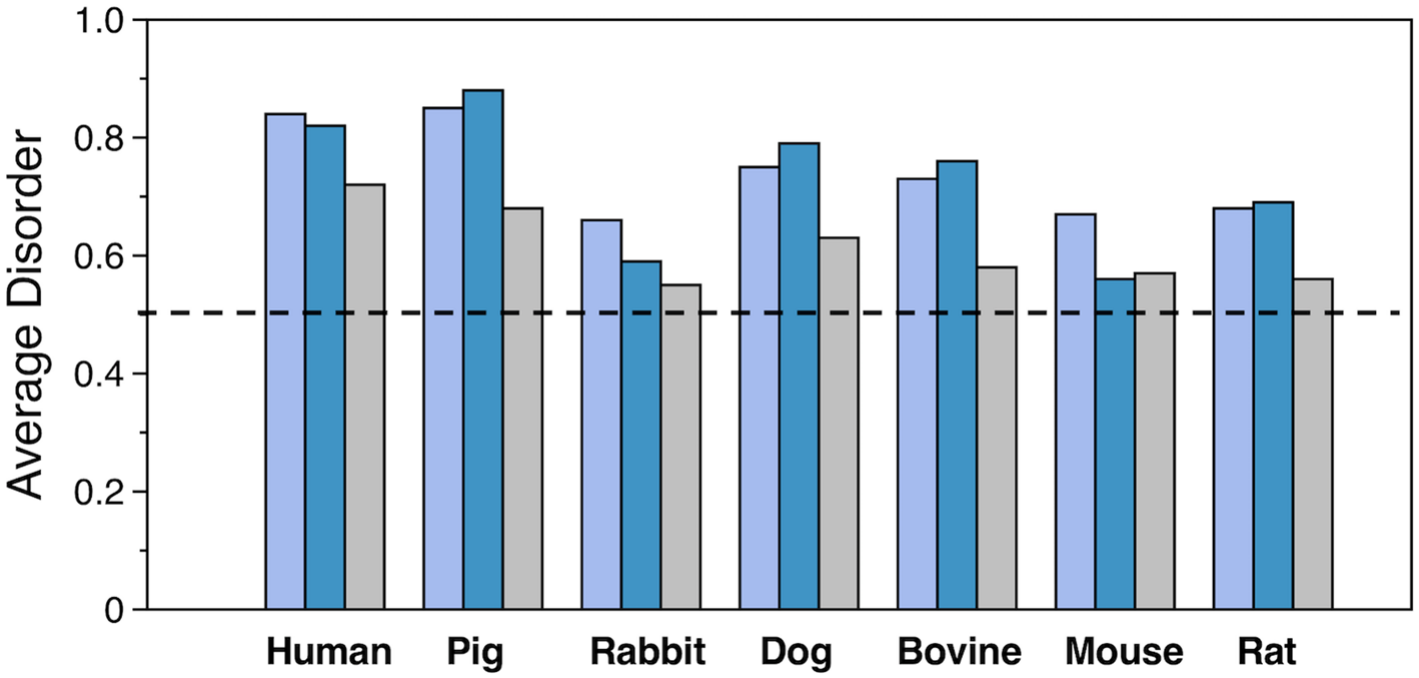
Evaluation of the intrinsic disorder propensity of various mammalian protein C amino acid sequences. Shown are the average disorder scores for the sequences of the activation peptide (purple), the activation peptide and the basic linker that connects it to the EGF2 domain (blue), and the P12-P12′ residues (gray). Scores were calculated from analysis of the entire amino acid sequence as described in Methods.

To better evaluate the evolutionary conservation of the disorder propensity in the activation peptide region, we constructed profiles of all mammalian amino acid sequences of protein C currently deposited in the UniProt database. After analyzing the entire sequence with the VSL2 algorithm, we calculated the average disorder score for the activation peptide with or without the basic linker that connects it to the EGF2 domain and the score for the sequence of the P12-P12′ residues (Fig. 3). Even though the activation peptide region in all protein C sequences tends to be disordered, we found moderate differences in their disorder disposition. The most pronounced level of disorder in the three regions of interest was observed in protein C from human and pig, while the lowest disorder propensity was found in the zymogen from rabbit, mouse and rat (Fig. 3). Because the binding of disordered regions is often accompanied by unfavorable entropic cost^67^, it remains to be determined whether mammalian sequences that have greater disorder disposition in their activation peptide region are activated by thrombin at a slower rate.

### Activation of protein C mutants by thrombin

**Figure 4:**
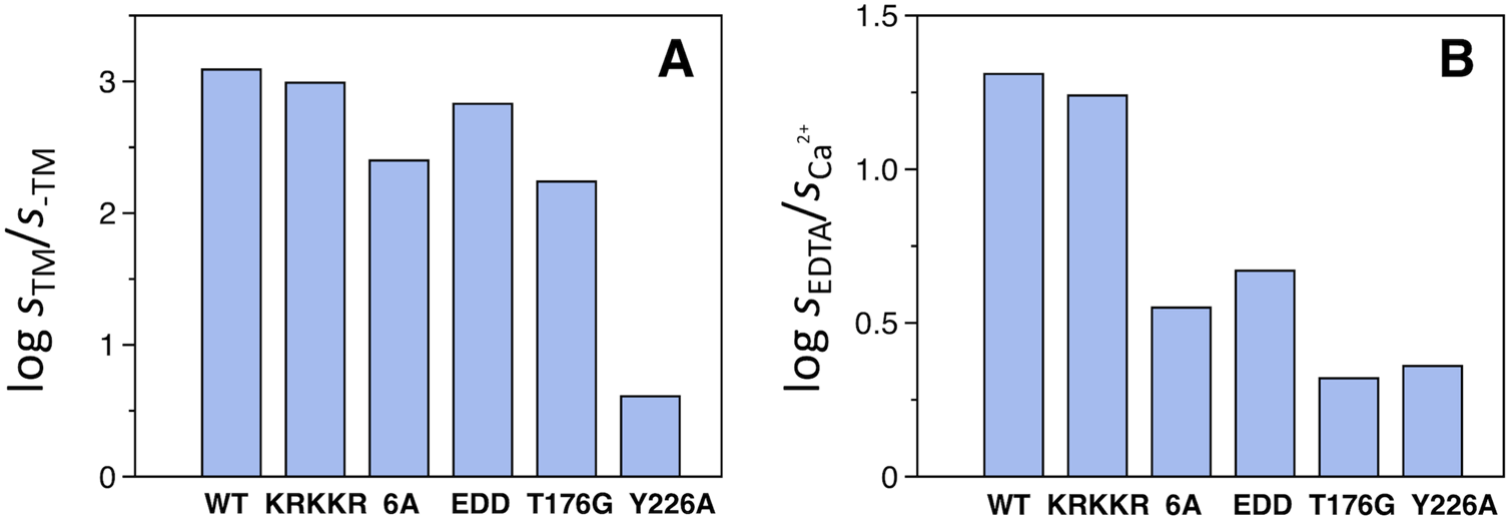
Activation of protein C variants by thrombin. Shown are the values of s = *k*_cat_/*K*_m_ measured **(A)** in the presence and absence of thrombomodulin (TM), and **(B)** in the presence of EDTA and CaCl_2_. Experimental conditions were: 20 mM Tris, pH 7.5, 145 mM NaCl, 0.1% PEG 8,000 at 37 °C. The buffer used for the reactions shown in panel A was supplemented with 10 mM CaCl_2_.

To understand how the clustering of acidic and basic residues in the activation peptide of protein C influences the rate of activation by thrombin, we expressed two mutants where the majority of charged residues were neutralized by Ala replacement. The 6A mutant (D158A/E160A/D161A/E163A/D164A/D167A) features all acidic amino acids replaced by Ala and the KRKKR mutant (K146A/R147A/K150A/K151A/R152A) has five basic amino acids replaced in the linker region. Residues K156 and R157 are part of the dipeptide that is proteolytically removed by a furin-like proprotein convertase^71^ during the secretion process and were left intact. Neutralization of the basic cluster of residues has no effect on the activation rate by thrombin under all conditions tested, i.e., with and without Ca^2+^ or thrombomodulin (Table 1). In contrast, substitution of the acidic residues in the 6A mutant enhanced the rate of activation 12-fold in the presence of Ca^2+^, but only marginally (twofold) in the absence of Ca^2+^ and in the presence of thrombomodulin (Table 1 and Fig. 4). Enhanced activation rates were also observed with the EDD mutant (E160A/D167A/D172A), which was characterized previously in the GLA-domainless form^25^. The results indicate that the acidic cluster contributes to the inhibitory effect of Ca^2+^ on activation of wild type protein C in the absence of thrombomodulin. It is possible that some of the acidic residues in the activation peptide assume a conformation that “cages” R169 in the scissile bond in the presence of Ca^2+^^25^, thereby restricting accessibility of this residue to thrombin. Alternatively, or in addition to the foregoing mechanism, neutralization of six acidic residues may reduce flexibility and disorder of the activation peptide and the entropic cost associated with the binding interaction with thrombin.

**Figure 5:**
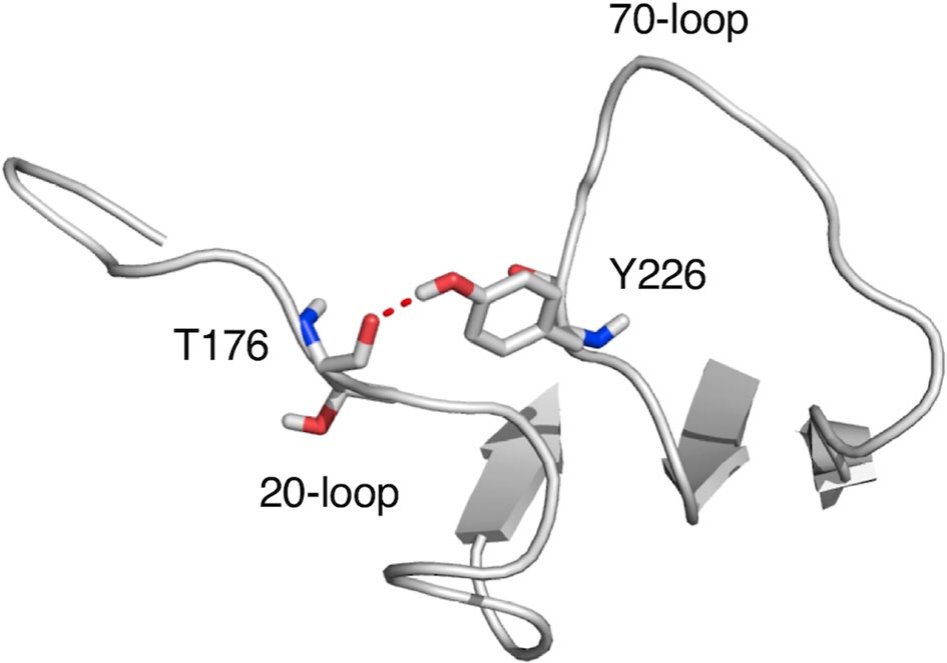
The H-bond between T176 and Y226 in the crystal structure of activated protein C^16^ connects the 20- and 70- loops of the protease domain. These residues correspond to T22 and Y71 in the chymotrypsin numbering. Image drawn with PyMOL (www.pymol.org).

Several studies have shown that binding of Ca^2+^ and thrombomodulin affects the conformation of the activation peptide of protein C^25,40,41^. In the absence of a structure for protein C, it is unclear how these interactions are transduced allosterically to the region around the thrombin cleavage site at R169. The locale for Ca^2+^ binding is the 70-loop of the protease domain of protein C^16,40,52^. Thrombomodulin binds to residues located in the 30-, 60- and 70-loops^72–75^. These loops are numbered according to alignment of the protease domain of protein C with chymotrypsin and residues numbered according to this nomenclature are shown in parenthesis. The crystal structure of activated protein C^16^ shows the 70-loop in close proximity to the 20-loop close to the site of activation at R169 (R15) and a strong H-bond forms between the backbone O atom of T176 (T22) and the side chain of Y226 (Y71) (Fig. 5). The mutant Y226A drastically compromises the ability of thrombomodulin to enhance the rate of protein C activation by thrombin (Table 1 and Fig. 4). The 1,500-fold increase observed for wild type is reduced to only fourfold in the mutant. The Y226A mutation also alleviates the inhibitory effect of Ca^2+^ in the absence of thrombomodulin. In the wild type, Ca^2+^ inhibits the rate of activation 20-fold but in the mutant the effect is reduced to twofold (Table 1 and Fig. 4). We propose that the ineffective activation of the Y226A mutant by the thrombin-thrombomodulin complex primarily arises from perturbation of the H-bond with T176; future experiments with other Y226 variants (*i.e.*, Y226F) should clarify how important the bulky benzyl ring is for the cofactor-dependent stimulation on the rate of activation.

The critical T176-Y226 interaction was also perturbed from the 20-loop side by introducing the T176G mutation. However, the T176G mutant has no effect on the thrombomodulin-mediated enhancement of protein C activation which remains as pronounced as in the wild type (Table 1 and Fig. 4). This is probably because Gly, just like any residue that is introduced at position 176, can still form an H-bond through its main chain O atom with the side chain of Y226 and preserve the structural connectivity between the 20- and 70-loops. On the other hand, the T176G mutation reduces the inhibitory effect of Ca^2+^ to about twofold relative to the rate measured in the presence of EDTA. Also, in the presence of Ca^2+^, the T176G variant is activated at a rate that is 11-fold faster than wild-type (Table 1 and Fig. 4). We propose that the alleviation of the Ca^2+^ inhibitory effect that is seen with the T176G mutant results from elimination of the rigid branched side chain which increases the flexibility of neighboring residues in the 20-loop. Branched side chains, such as that of Thr, are known to restrict the flexibility of the main chain torsion angles, while Gly, which lacks a side chain, has the opposite effect. Future mutagenesis experiments should clarify to what extent a branched side chain at position 176 is necessary in mediating the Ca^2+^ inhibitory effect through minimizing the conformational entropy of the 20-loop.

### Activation by factor Xa

**Figure 6:**
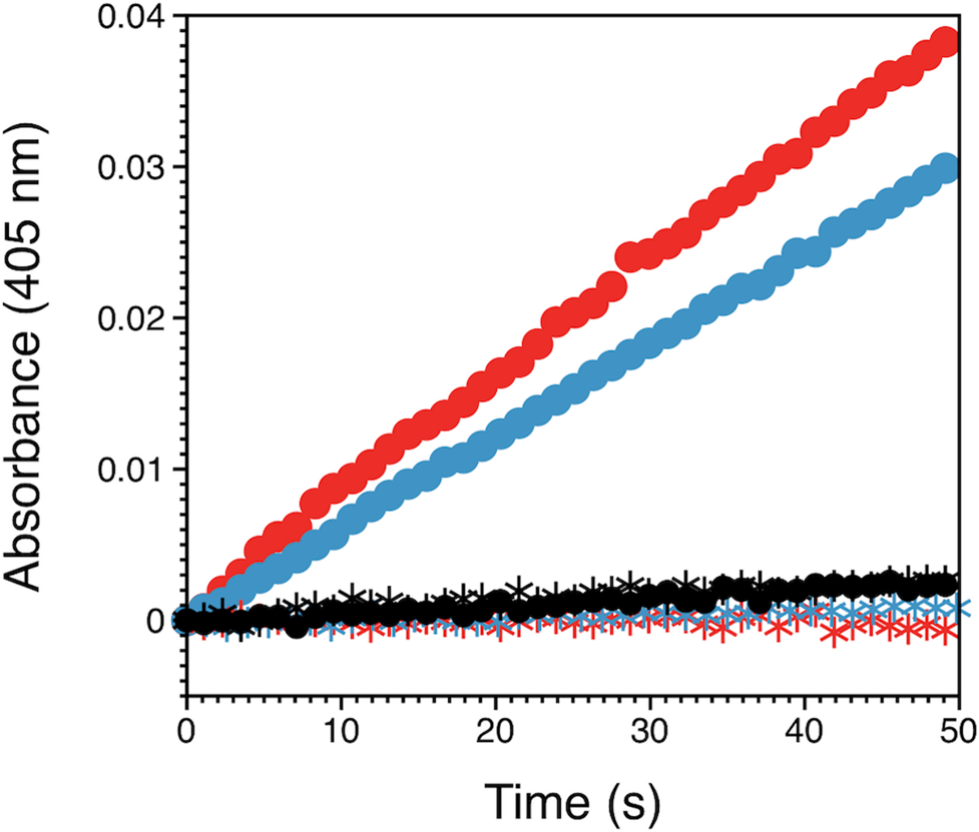
Activation of protein C variants by factor Xa. Constructs were incubated with 50 nM factor Xa for 90 min and the reaction was quenched with excess apixaban. Formation of activated protein C wild type (black circles), mutant 6A (red circles) and mutant EDD (blue circles) was quantified by monitoring the absorbance at 405 nm that resulted from cleavage of the chromogenic substrate S-2366. The *k*_cat_/*K*_m_ values for the FXa catalyzed activation of 6A and EDD are 1.4 ± 0.2 mM^−1^ s^−1^ and 0.57 ± 0.05 mM^−1^ s^−1^, respectively. Control experiments without addition of factor Xa to the reaction mixture are shown by asterisks for protein C wild type (black), mutant 6A (red) and mutant EDD (blue). Experimental conditions were: 20 mM Tris, pH 7.5, 145 mM NaCl, 10 mM CaCl_2_, 0.1% PEG 8,000, 200 μM phospholipids, 250 nM hirudin at 37 °C.

We have previously shown that a GLA-domainless protein C variant carrying the triple EDD mutation spontaneously autoactivates over a slow time scale^25^. The three acidic residues in protein C have a direct counterpart in the zymogen prethrombin-2 where they structurally “cage” R15. Once replaced to Ala, the site of activation at R15 is exposed to solvent and prethrombin-2 rapidly converts to thrombin by autoactivation^49,76^. Screening of the site of activation from solvent may be a general strategy for protection from unwanted proteolysis, especially among zymogens with modular assembly. In addition to prothrombin and protein C, plasminogen assumes a closed form stabilized by intramolecular interaction of the activation domain with kringles that keeps the zymogen in an activation-resistant conformation^77^. Binding of kringles to fibrin clots and cell-surface receptors induces a transition to an open form that can be cleaved and converted to plasmin by physiological activators. The activation peptide of factor X also appears to play a protective role against autoactivation. Rudolph et al.^61^ have reported that deletion of residues 137–183 from the activation peptide of factor X produces a mutant with increased propensity for intermolecular activation in the presence of membrane surfaces. Importantly, the mutant becomes susceptible to activation by thrombin^61^, contrary to what it is observed for wild type under physiological conditions. We therefore examined the possibility that perturbation of the acidic residues in the activation peptide of protein C could introduce specificity toward proteases other than the physiological activator thrombin. Indeed, the protein C mutant 6A is activated by factor Xa at a significant rate under conditions where wild type protein C is not (Fig. 6). A similar effect is also observed with the EDD mutant.

## Discussion

In the present study we have employed bioinformatic analyses to evaluate the disorder profiles of the vitamin K-dependent zymogens that share the same modular domain assembly as protein C and contain an activation peptide that is proteolytically removed during activation. The activation peptide of protein C is predicted to be intrinsically disordered (Fig. 2) and to an extent that is more pronounced than in zymogens like factors IX and X. The disorder arises from the large density of acidic residues within the short activation peptide. Replacement of these acidic residues produces a more ordered activation domain that is less sensitive to the inhibitory effect of Ca^2+^ and is also cleaved by factor Xa in addition to the physiological activator thrombin. Effects observed for factor X^61^ may have a similar molecular origin. Removal of residues in the activation domain of this zymogen introduces specificity toward thrombin and also a tendency to autoactivate, possibly through ordering of the domain. It is also notable that, unlike protein C, the activation peptides of factors IX and X^60,61^ are glycosylated and that this post-translational modification may be needed to protect the scissile bond from proteolysis from non-physiological activators.

The combined replacement of all basic residues in the linker that connects the activation peptide with the EGF2 domain in protein C has no significant effect on the rate of activation by thrombin. Some of the residues in this linker are known to have a moderate influence on the proteolytic processing of the K156-R157 dipeptide by a furin-like proprotein convertase^71^. A recent study has documented an important role for K150 and K151 in the anticoagulant and cytoprotective functions of activated protein C^78^. In contrast to the basic cluster, the 6A mutant carrying substitutions in six acidic residues in the activation domain features a reduction in the inhibitory effect of Ca^2+^ and a modest enhancement of the effect of thrombomodulin on the rate of thrombin activation. The results are consistent with previous studies where mutations of D167 and E160 in the activation peptide and D172 immediately downstream to the site of cleavage at R169 produced single, double and triple mutants activated more rapidly by thrombin^25,39,50,51^. While part of the rate-enhancing effect that ensues from these mutations might result from attenuating the level of electrostatic repulsion between protein C and negatively charged residues that rim the active site of thrombin^79^, we believe that the acidic residues in the activation peptide primarily protect protein C activation in the absence of thrombomodulin by hindering access to the scissile bond. The effect is similar to what has been reported for prethrombin-2^25,49^. The relevant similarity between the two proteins in this region is demonstrated by the sequence ^311^**E**LLESYI**D**G**R**IV**E**^323^ in prethrombin-2 and ^160^**E**DQEDQV**D**P**R**LI**D**^172^ in protein C^25,49^, where the acidic residues caging R320 or R169 in the scissile bond are in bold. An alternative explanation may also be provided by the reduced disorder in the activation domain caused by the 6A mutation. Interactions involving highly disordered regions are usually energetically unfavorable due to large entropy costs associated with formation of a productive complex^67^. The two effects, i.e., caging of R169 and disorder in the activation domain, are not mutually exclusive and may cooperate in reducing cleavage of protein C by thrombin in the absence of thrombomodulin. The acidic residues in the activation domain targeted in this study may also play an important role in preventing non-physiological activation. The 6A and EDD mutants are activated by factor Xa at a significant rate, unlike wild type (Fig. 6). The results echo similar observations with coagulation factor X, where deletion in the activation peptide results in constructs that can be activated by thrombin, unlike wild type, and are also capable of autoactivation as observed in coagulation FVII that lacks an activation peptide^61^.

The results reported here support the new paradigm recently emerged for protein C activation where thrombomodulin acts as a dual cofactor that utilizes two end-points for its allosteric effect, i.e., the catalytic Ser of thrombin^44^ and the Arg residue at the site of cleavage of protein C^25^. Other residues of thrombin and protein C obviously participate in the allosteric effect of thrombomodulin, as suggested by several groups^24,29,34–38^, but they do so by eventually altering these two end-points.

## Methods

### Evaluation of intrinsic disorder propensity

The following amino acid sequences were downloaded from the UniProt database with their respective UniProt IDs shown in brackets: human factor IX [P00740], human factor X [P00742], and protein C from human [P04070], rabbit [Q28661], bovine [P00745], dog [Q28278], pig [Q9GLP2], rat [P31394] and mouse [P33587]. Each amino acid sequence was evaluated for its propensity for intrinsic disorder with the VSL2^68^, VLXT^69^ and VL3^70^ algorithms from the PONDR family of predictors. In all cases, we only considered the sequences of the mature proteins starting from the first amino acid in the GLA domain. Where applicable, after evaluating the intrinsic disorder propensity of the entire amino acid sequence, we calculated the average disorder score for the sequences around the activation peptide as the ratio of the disorder score sum over the total number of residues.

### PCR mutagenesis, protein expression and purification

Quick-change lightning site-directed mutagenesis kit (Agilent Technologies) was used to introduce the mutations described in the results sections into the human protein C plasmid carrying a C-terminal HPC-4 tag. Plasmids were transfected into baby hamster kidney (BHK) cells using X-tremeGENE 9 DNA transfection reagent (Roche) according to a standard protocol supplied by the manufacturer. After incubation of 48 h, selection of stably expressing clones was initiated by incubating the transfected cells with 1 mg/mL geneticin and expression of stably selected clones was verified by western blotting using the HPC-4 antibody. Stably selected clones were gradually expanded and transferred into large cell factories. Protein C variants were initially purified by immunoaffinity chromatography using a resin that was coupled with the HPC-4 antibody as described for prethrombin 1^29^. After the immunoaffinity chromatography step, the sample was diluted to achieve a final NaCl concentration below 50 mM and the protein was loaded onto a 1 mL Q-sepharose Fast-Flow (GE Healthcare) column attached through its top to a 1 mL HiTrap heparin column (GE healthcare) equilibrated with 20 mM Tris, pH 7.5, 50 mM NaCl, and 10 mM EDTA. Then the heparin column was detached and the protein was eluted from the Q-sepharose Fast-Flow column using a 0.05–1 M NaCl gradient. Lastly, the protein was purified by size-exclusion chromatography using a pre-packed superdex 200 column (GE Healthcare) equilibrated with 20 mM Tris, pH 7.5 and 145 mM NaCl.

### Kinetic assays

Activation of protein C variants was monitored using a discontinuous assay under pseudo-first order conditions where the concentration of substrate was maintained below the *K*_m_ value. Reactions initiated by thrombin (1–150 nM) were measured in the presence of 10 mM CaCl_2_ with or without rabbit thrombomodulin (50–200 nM) or in the presence of 5 mM EDTA. The factor Xa assays were conducted in the presence of 200 μM phospholipids (75% phosphatidylcholine and 25% phosphatidylserine) and included 250 nM hirudin in order to exclude any activity from possible contamination with thrombin. Reactions with thrombin were stopped at specific time intervals with excess hirudin, while those with factor Xa were quenched with the specific inhibitor apixaban (MedChemExpress). Formation of activated protein C at given time intervals was quantified from the cleavage of the chromogenic substrate S-2366 (Diapharma) by monitoring the absorbance at 405 nm. The *k*_cat_/*K*_m_ value was obtained after fitting the initial velocities to an exponential equation. Assays were performed at least in duplicates with standard errors lower that 5%. All measurements were conducted under experimental conditions: 20 mM Tris, pH 7.5, 145 mM NaCl, 0.1% PEG 8,000 at 37 °C. Thrombin was purified and activated as described previously^29^. Human factor Xa was purchased from Haematologic Technologies Inc.

**Table 1.** Values of *k*_cat_/*K*_m_ (mM^−1^ s^−1^) for the activation of wild-type and mutant protein C by thrombin

	− TM + CaCl_2_	+ TM + CaCl_2_	− TM + EDTA
Wild type	0.18 ± 0.01	220 ± 20	3.7 ± 0.3
KRKKR	0.19 ± 0.04	186 ± 2	3.3 ± 0.6
6A	2.2 ± 0.3	550 ± 50	8.1 ± 0.9
EDD	2.3 ± 0.1	1,590 ± 40	11 ± 1
T176G	2.0 ± 0.3	350 ± 30	4.2 ± 0.6
Y226A	0.8 ± 0.1	3.3 ± 0.1	1.9 ± 0.5

Experimental conditions: 20 mM Tris, pH 7.5, 145 mM NaCl, 0.1% PEG8000 with 10 mM CaCl_2_ or 5 mM EDTA at 37 ºC. TM, Thrombomodulin

## Data Availability

Recombinant reagents and data presented in this study are available from the corresponding author upon reasonable request.
